# Effect of pH and (Y, Ag) co-doping on the antibacterial and toxicity properties of ZrO_2_ nanoparticles

**DOI:** 10.1039/d5na00649j

**Published:** 2025-09-30

**Authors:** Mehedi Hasan Jasim, Md. Iqbal Hossain, Yasfir Mahmud, A. K. M. Ahsanul Habib, Moumita Tasnim Meem

**Affiliations:** a Department of Materials Science & Engineering, Rajshahi University of Engineering & Technology Rajshahi Bangladesh 1813022@student.ruet.ac.bd

## Abstract

Nowadays, the effective bacterial inhibition and minimal toxicity properties of ZrO_2_ nanoparticles are creating opportunities in various applications, including biomedical, dental, antimicrobial coatings, packaging, anticancer and pesticide applications. Therefore, researchers are employing various approaches to enhance the antibacterial activity and reduce the toxicity of ZrO_2_ nanoparticles. In this study, undoped or pure ZrO_2_ nanoparticles were synthesized at different pH levels (1, 3, 7, and 11) and Y and Ag were codoped in different doping concentrations (1%, 2%, and 3%). A sucrose-assisted sol–gel method was used to synthesis both undoped and codoped ZrO_2_ nanoparticles. The main objective of this research was to analyze the effect of pH and codoping on the antibacterial and toxicity properties of ZrO_2_ nanoparticles. The synthesized particles were extensively characterized by X-ray diffraction (XRD), scanning electron microscopy (SEM), energy-dispersive X-ray spectroscopy (EDX), and Fourier transform infrared (FTIR) spectroscopy. XRD analysis confirmed the existence of ZrO_2_ nanoparticles with a tetragonal structure at low pH (1 and 3), a monoclinic structure at higher pH (7 and 11) and a multiphase structure after codoping. The crystallite size of undoped ZrO_2_ particles increased with pH, from 8 nm at pH 1 to 20 nm at pH 11, indicating enhanced crystallinity at higher pH levels. Also, the crystallite size was 12.05, 14.81, and 11.95 nm at 1%, 2%, and 3% doping concentrations, respectively. SEM analysis revealed that the average particle size increased with increasing pH from 31.956 nm to 63.653 nm. The particle size was 34.90 nm, 48.66 nm, and 40.23 nm for 1%, 2% and 3% doped samples, respectively. The shape of the particles was irregular and mostly spherical, rod-like, and platelet-like with agglomeration. The EDX data determined the elemental composition of the particles, which indicated the successful doping with Y and Ag. The FTIR data revealed the existence of O–H, Zr–O, and other metal–oxygen vibrations in the synthesized materials. The antibacterial activity of undoped and codoped ZrO_2_ nanoparticles was evaluated against *Escherichia coli* (Gram-negative) and *Staphylococcus aureus* (Gram-positive) using the disk diffusion method. The result showed that a superior antibacterial activity was observed at pH 11 for undoped particles and 3% Y–Ag codoped particles. The toxicity of the undoped and codoped ZrO_2_ nanoparticles was inspected using the brine shrimp lethality assay, which showed that the material was less cytotoxic at lower pH (1 and 3), and the toxicity increased with increasing doping concentration. Increasing pH and doping concentration both enhanced the antibacterial and cytotoxic activities, making the material suitable for antimicrobial coatings, packaging, and textiles, with potential for pesticide or anticancer applications pending further evaluation.

## Abstract 2

Y–Ag co-doped ZrO_2_ nanoparticles show enhanced anti-bacterial activity with increasing pH and doping, while their toxicity remains low at acidic pH, highlighting potential for eco-friendly anti-microbial applications.

## Graphical abstract

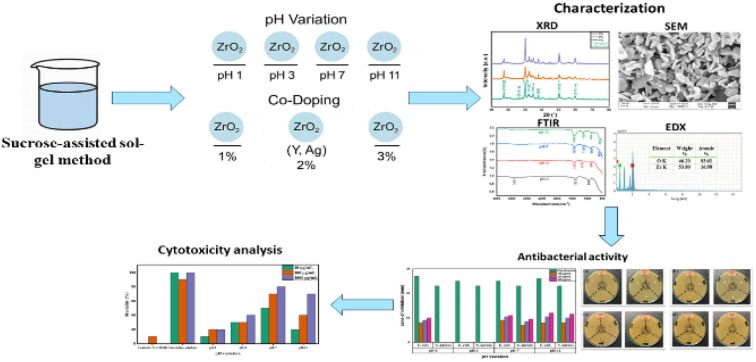

## 1. Introduction

In recent years, nanomaterials have grabbed the attention in the field of modern science and technology, especially in the biomedical field, including antibacterial activity, toxicity activity, and drug delivery.^1–3^ Their physicochemical properties and high surface area-to-volume ratio make them ideal candidates in this field.^4^ Among metal oxide nanoparticles, zirconia (ZrO_2_) has drawn interest because of its mechanical strength,^5^ thermal stability,^6^ and biocompatibility,^7^ positioning it as a possible material for cytotoxic and antibacterial applications.^8,9^ Research has revealed that ZrO_2_ nanoparticles can prevent bacterial growth *via* cell membrane disruption and reactive oxygen species (ROS) generation.^10^ Furthermore, their cytotoxic characteristics are absolutely vital for evaluating biocompatibility in biomedical applications.^7,11^

The properties of ZrO_2_ nanoparticles are largely defined by their synthesis conditions, and pH is a major element affecting their growth. Changes in pH can affect the particle size, surface charge, and crystallographic phase, all of which influence their stability and interaction with the biological environment.^12–14^ The particle size increases with increasing pH due to a change in reaction kinetics and stronger surface charge repulsion at higher pH.^15^ The crystallographic phase of the nanoparticles also changes with pH variation, as elevated hydroxyl ion concentrations drive the phase transformation from the metastable tetragonal structure to a thermodynamically favored monoclinic structure.^12,16^ Variations in pH also affect ion release, which may change the cytotoxic and antibacterial properties of nanoparticles.^14,17^

The combination of silver (Ag) and yttrium (Y) as codopants in ZrO_2_ nanoparticles overcomes important barriers related to achieving effective antibacterial properties without compromising material biocompatibility.^18,19^ Ayanwale *et al.*^20^ achieved inhibition zones of 15–22 mm against *E. coli*, *S. aureus*, and other pathogens using ZrO_2_–Ag_2_O nanoparticles. Sredojević *et al.*^21^ demonstrated that Ag-doped ZrO_2_ functionalized with dihydroquercetin (DHQ) displayed low cytotoxicity to healthy human cells (MRC-5) and cancer cells (HeLa) at concentrations up to 0.50 mg mL^−1^. In another study, Alzahrani *et al.*^22^ showed that the yttria-stabilized zirconia nanoparticles induce the dose- and time-dependent apoptosis of human skin keratinocytes (HaCaT cells). Exposure to 60 μg mL^−1^ for 48 hours reduced cell viability to 43% *via* reactive oxygen species (ROS) generation, mitochondrial membrane depolarization, and caspase-3 activation. However, the relationship between yttrium doping and silver enhances material longevity by exhibiting extended biocidal activity by combining silver's antibacterial properties with yttrium's redox buffering characteristics, which improves cell-friendly reactions, thus enabling extended-period use in implanted medical devices.^23^

Codoping with Y and Ag was performed at 1%, 2%, and 3% concentrations to systematically evaluate the dose-dependent effects on the antibacterial activity and cytotoxicity. The selection of these specific doping concentrations was based on previous studies, which suggested that low Ag doping (≤3%) effectively enhances antimicrobial properties without inducing significant cytotoxicity.^24^ If Ag is present in amounts greater than 3%, it may generate too many ROS, causing toxicity to mammalian cells.^25^ Meanwhile, Y doping stabilizes the ZrO_2_ lattice, preventing undesirable phase transformations while maintaining biocompatibility.^26^ A pH of 11 was chosen for the codoped synthesis because alkaline conditions favor the stabilization of the tetragonal phase of ZrO_2_, promote better dopant incorporation, and help maintain a nanoscale particle size, all of which are essential for maximizing the functional benefits of Y and Ag codoping^27^ This strategic combination of controlled pH and optimized dopant levels provides a new pathway for designing ZrO_2_-based nanomaterials with superior biomedical performance.

Although extensive research has been conducted on ZrO_2_ nanoparticles, comprehensive studies evaluating the combined effects of codoping and pH conditions on their antibacterial and cytotoxic behavior remain limited. Most existing studies have investigated the individual impacts of Ag and Y doping or the role of pH, leaving a significant gap in understanding their synergistic influence.^28^ Furthermore, there is a lack of systematic studies exploring how varying pH environments affect the biological properties of codoped ZrO_2_ nanoparticles. Given the growing demand for nanomaterials that exhibit enhanced antimicrobial activity with minimal toxicity, a thorough understanding of the interplay between codoping elements and synthesis conditions is essential.^17,29,30^ This study aims to bridge this gap by systematically analyzing the influence of pH variations and (Y, Ag) codoping on the antibacterial and cytotoxic properties of ZrO_2_ nanoparticles, thereby providing valuable insights into their potential biomedical applications.

In this article, the synthesis of undoped ZrO_2_ nanoparticles at different pH and Y–Ag codoped ZrO_2_ nanoparticles using a sucrose-assisted sol–gel method and their improved antibacterial and cytotoxic activities were explored. The samples' characteristics were analyzed *via* X-ray diffraction (XRD), scanning electron microscopy (SEM), energy-dispersive X-ray spectroscopy (EDX), and Fourier transform infrared (FTIR) spectroscopy. At the same time, the nanoparticles' antibacterial activities were checked against *Escherichia coli* (Gram-negative) and *Staphylococcus aureus* (Gram-positive) using the disk diffusion method at varying concentrations. The toxicity of ZrO_2_ nanoparticles was inspected using the brine shrimp lethality assay.

## 2. Experimental section

### 2.1. Materials

Materials utilized in this study are as follows: (1) precursor: zirconium nitrate, Zr(NO_3_)_4_ (CAS 13746-89-9, assay 99.9%, Merck-India); (2) dopant precursors: silver nitrate, AgNO_3_ (CAS 7761-88-8, assay 99.9%, Merck-India) and yttrium(iii) nitrate hexahydrate, Y(NO_3_)_3_·6H_2_O (CAS 13494-98-9, assay 99.9%, Merck-India); (3) reducing agent: sucrose, C_12_H_22_O_11_ (CAS 57-50-1, assay 99.9%, Merck-India); (4) sodium hydroxide, NaOH (CAS 1310-73-2, assay 99.9%, Merck-India); (5) hydrochloric acid, HCl (CAS 7647-01-0, assay 99.9%, Merck-India); (6) bacterial strains for antibacterial evaluation: Gram-positive bacteria *Staphylococcus aureus* (*S. aureus*) and Gram-negative bacteria *Escherichia coli* (*E. coli*); (7) toxicity analysis reagents: brine shrimp (*Artemia salina*) eggs, vincristine sulphate (CAS 2068-78-2, standard purity) and dimethyl sulfoxide (DMSO, CAS 67-68-5, assay 99.9%, Merck-India); and (8) distilled water as the synthesis medium. All the materials were used without any further purification.

### 2.2. Synthesis of undoped and codoped ZrO_2_ nanoparticles

**Fig. 1:**
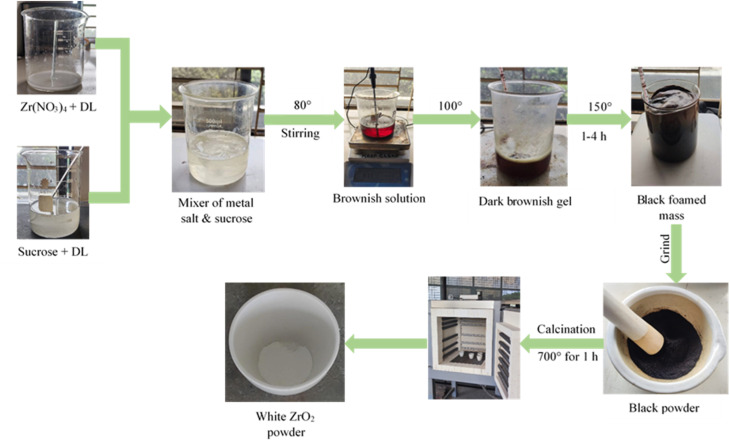
Schematic diagram of the synthesis of ZrO_2_ nanoparticles.

First, 6.786 g (0.02 mole) of Zr(NO_3_)_4_ was dissolved in 20 mL of deionized water. The sucrose solution was prepared separately by combining 123 g (0.36 mole) of sucrose with 90 mL of deionized water. After the two solutions were combined and constantly agitated for 10 minutes, the pH of the resulting homogeneous solution was adjusted to 1, 3, 7, or 11 by adding controlled amounts of HCl (1 M) and NaOH (1 M) dropwise. The sucrose-to-metal ion ratio was maintained at 18 : 1. The ions were reacted with sucrose by heating at 80 °C for 2 hours on a hot plate while continuously stirring. A brownish solution was obtained. The solution was then dehydrated by heating on the hot plate over 100 °C, and this process was continued until the solution turned into a viscous dark-brownish gel. For complete dehydration, this gel was subsequently baked at 150 °C for 1–4 hours in an oven, producing a black foamed mass. This mass was then ground into a powder and placed in a furnace at 700 °C for 1 hour to remove any carbon present. During the 1-hour calcination process, carbon was oxidized from the black precursor powder, forming a white-colored powder. Fig. 1 illustrates the synthesis process of ZrO_2_ nanoparticles. For doping, just 1%, 2%, or 3% dopants were added after the zirconium nitrate solution, and the remainder of the technique was the same as that for producing undoped ZrO_2_ nanoparticles.

### 2.3. Characterization of the nanoparticles

ZrO_2_ nanoparticles were initially characterized by a RIGAKU SmartLab (Japan) X-ray diffractometer equipped with a Cu-Kα irradiation source (wavelength: 1.5406 Å) at 40 kV and 30 mA. XRD patterns were obtained in the 2*θ* range from 10° to 80° at a scanning rate of 20° min^−1^. The microstructure and surface morphology of undoped and codoped ZrO_2_ nanoparticles synthesized at different pH levels were analyzed by scanning electron microscopy using ZEISS-EVO 18 (UK). EDS spectra were obtained to verify the elemental composition of the nanoparticles. FTIR analysis was performed using Thermo Scientific's Nicolet iS50R (USA) to identify the vibrational peaks of the nanoparticles. The spectra were obtained in the 400–4000 cm^−1^ region with a 4 cm^−1^ resolution.

### 2.4. Antibacterial activity

The antibacterial activity of the ZrO_2_ NPs was assessed using the well disc diffusion method. The experiment was performed against references *Escherichia coli* (MTCC 443, Gram-negative) and *Staphylococcus aureus* (MTCC 96, Gram-positive) obtained from the Microbial Type Culture Collection (MTCC), India. An inoculum size of 10^5^ cells per mL was applied to the Mueller–Hinton agar (MHA, HiMedia, India). In brief, 20 mL of MHA was dispensed into Petri dishes and permitted to solidify. Subsequently, 6-mm-thick sterile discs were accurately positioned on Petri dishes. Different concentrations of ZrO_2_ nanoparticles (100, 200, and 300 μg mL^−1^) were applied to each disk. Ciprofloxacin (5 μg per disc) was used as a positive control, and 50% ethanol served as a negative control. All plates were incubated at 37 °C for 24 hours, and the corresponding inhibition zones were measured. The same procedure was applied for both undoped and codoped samples.

### 2.5. Toxicity analysis

This study employed a simplified methodology for the brine shrimp lethality test, as proposed by Meyer *et al.*^31^*Artemia salina* eggs (cysts) were used because of their high hatchability and easy availability from aquarium pet suppliers. This test was called toxicity analysis instead of cytotoxicity analysis because it was done on brine shrimp. The term cytotoxicity is only used when the test is performed on mammals. A salt solution (prepared with 2–4% iodine-free table salt in distilled water) was used to incubate the cysts, allowing larvae (nauplii) to hatch within 24 hours under constant aeration and illumination. Freshly hatched nauplii were collected and introduced into solutions containing ZrO_2_ nanoparticles at concentrations of 10, 100, and 1000 μg mL^−1^. Both pH-varied and Y–Ag codoped ZrO_2_ nanoparticles were evaluated. Concentrations (10, 100, and 1000 μg mL^−1^) were selected based on standard ranges in brine shrimp lethality assays to evaluate dose-dependent effects from low (nontoxic) to high (potentially cytotoxic) levels, as per established protocols (Meyer *et al.*;^32^ Tabassum *et al.*^33^). This logarithmic progression allowed assessment across a broad spectrum while aligning with previous nanoparticle toxicity studies. Each test was performed in triplicate, with 10 nauplii placed in each test tube containing the nanoparticle solution. A contrast control group containing 1% DMSO (the nanoparticle dispersant) and vincristine sulfate (0.5 μg mL^−1^) was used as a positive control because it is a well-established cytotoxic drug frequently applied in brine shrimp lethality and cell-based toxicity assays.^32^ After 24 hours of exposure, the number of surviving nauplii was counted, and the percentage mortality was calculated using the following formula:
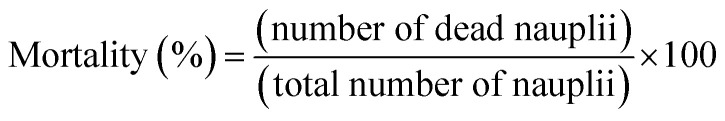


## 3. Results and discussion

### 3.1. XRD analysis

**Fig. 2:**
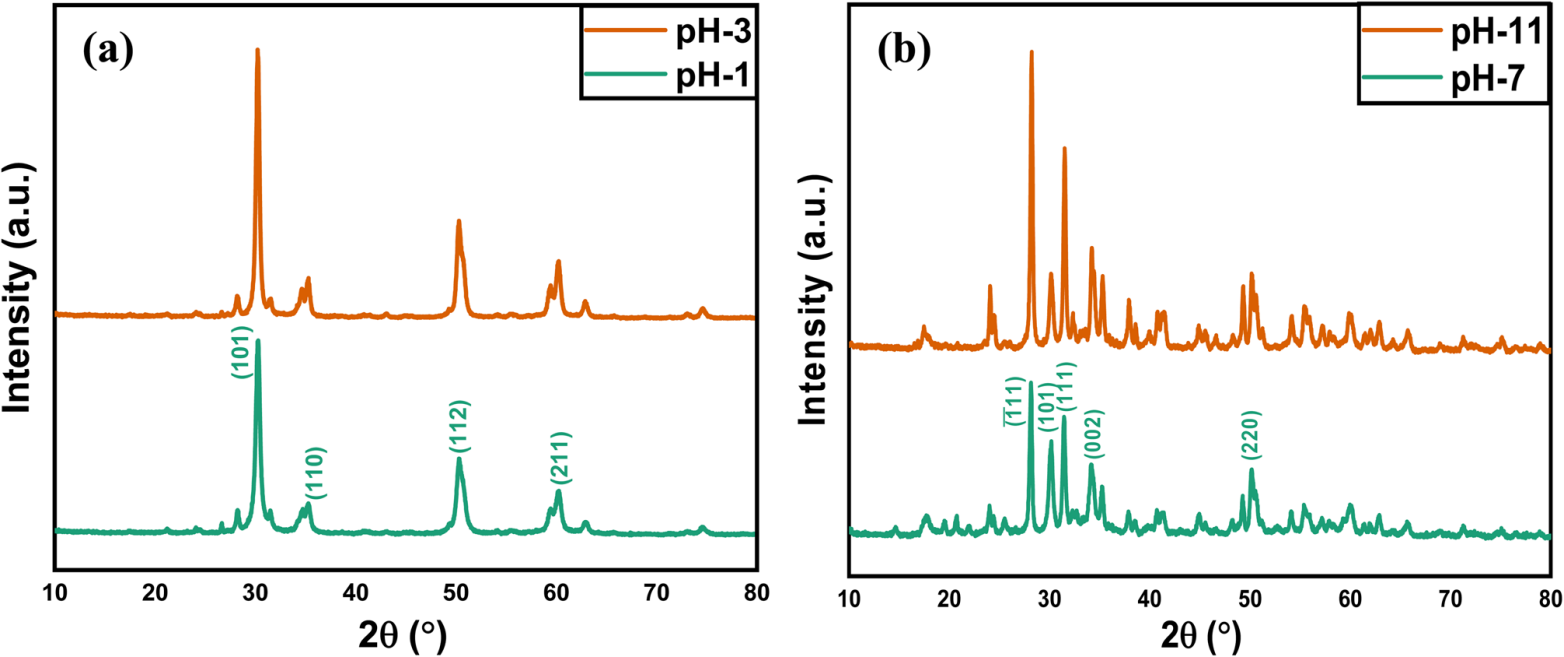
XRD patterns of undoped ZrO_2_ nanoparticles synthesized at (a) pH 1 and pH 3 and (b) pH 7 and pH 11.

The XRD graphs of the undoped ZrO_2_ nanoparticles synthesized at pH 1 and pH 3 are presented in Fig. 2(a). The diffraction peaks at 30.26°, 34.98°, 50.43°, and 60.04° correspond to the (101), (110), (112), and (211) planes, respectively, from the International Centre for Diffraction Data (ICDD) reference code 01-080-0784. The XRD pattern shows that the diffraction peaks correspond to the tetragonal structure and space group of *P*4_2_/*nmc*.

**Fig. 3:**
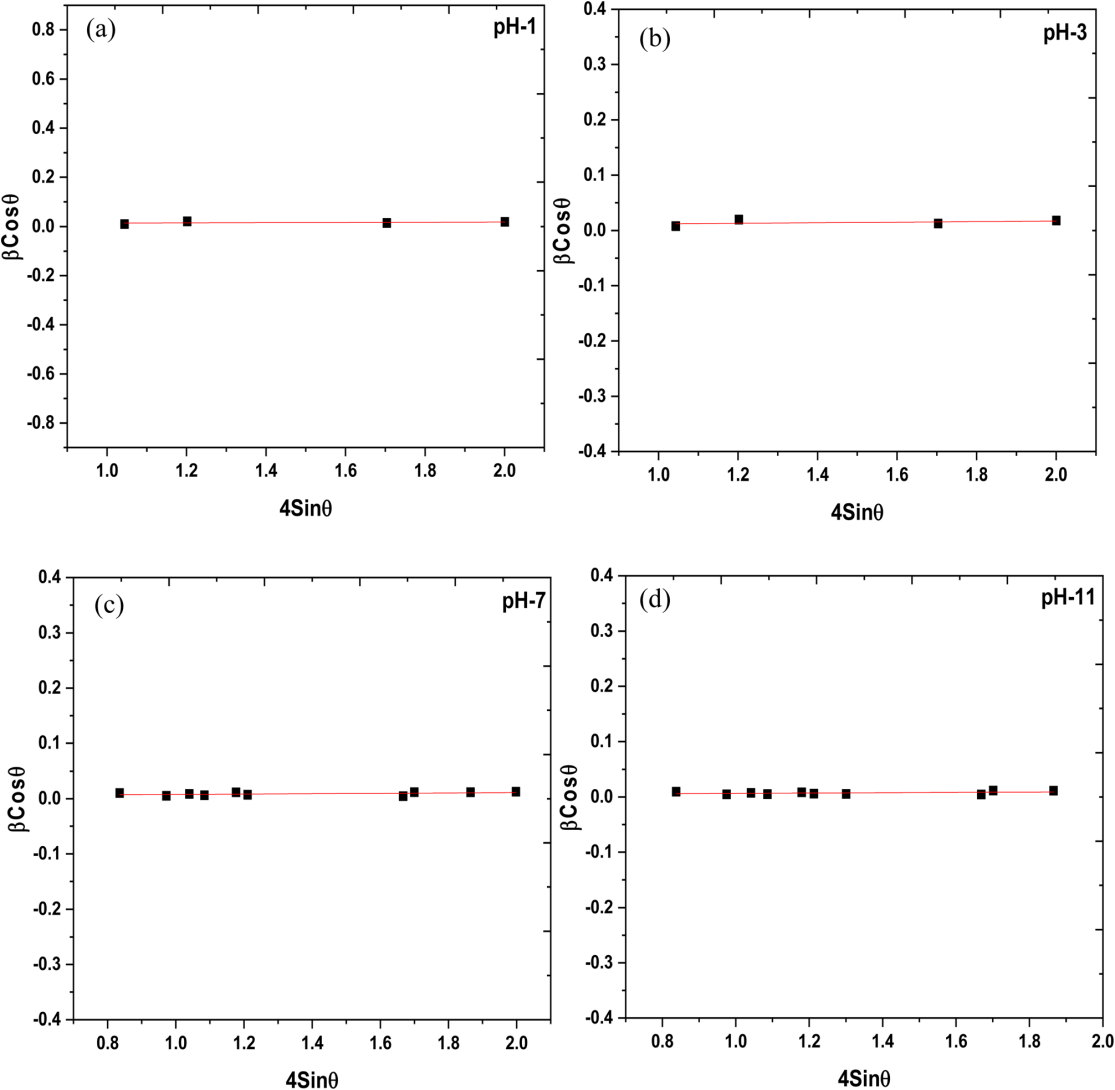
Williamson–Hall plots of undoped ZrO_2_ nanoparticles synthesized at (a) pH 1, (b) pH 3, (c) pH 7 and (d) pH 11.

The XRD graphs of the undoped ZrO_2_ nanoparticles synthesized at pH 7 and pH 11 are presented in Fig. 2(b). The diffraction peaks at 28.14°, 30.14°, 31.38°, 34.21, and 50.21° correspond to the (−111), (101), (111), (022), (220) planes, respectively, matching with the ICDD reference code 01-086-1451. The XRD pattern signifies that the diffraction peaks correspond to the monoclinic structure and space group of *P*2_1_/*c*. This work shows that the phase transformation of ZrO_2_ nanoparticles during the sucrose-assisted sol–gel process can be ascribed to the interaction of the pH-dependent solubility of zirconium hydroxide (Zr(OH)_*x*_O_*y*_), nucleation kinetics, and the stabilizing function of sucrose. While sucrose functions as a chelating agent, regulating the release of Zr^4+^ and therefore preventing particle growth, at low pH of 1 and 3, the high solubility of Zr(OH)_*x*_O_*y*_ generates Zr^4+^ ions, promoting rapid nucleation, stabilized by sucrose's chelation, which restricts crystallite growth and induces lattice distortions, resulting in high microstrain.^34^ Surface energy effects cause smaller crystals with stabilization in the metastable tetragonal phase (t-ZrO_2_) to arise.^35^ At high pH of 7 and 11, however, the higher concentration of OH^−^ ions accelerates hydrolysis and condensation, thereby encouraging the development of larger crystals, resulting in a larger crystallite size and a lower microstrain, which stabilizes the thermodynamically stable monoclinic phase (m-ZrO_2_).^34,36,37^Table 1 lists the average microstrain and crystallite size of the undoped particles. The following Williamson–Hall and Scherrer equations were used to calculate these parameters. Eqn (1) and (2), respectively, provide the Scherrer and Williamson–Hall equations. Table 1 indicates that the size of the crystallites increases as the pH increases. Fig. 3 shows the Williamson–Hall plots of ZrO_2_ nanoparticles synthesized at pH 1, pH 3, pH 7 and pH 11. Specifically, as the pH values increase, the crystallite size increases, whereas the microstrain decreases.1
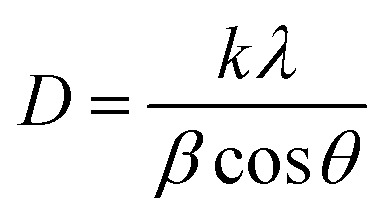
2
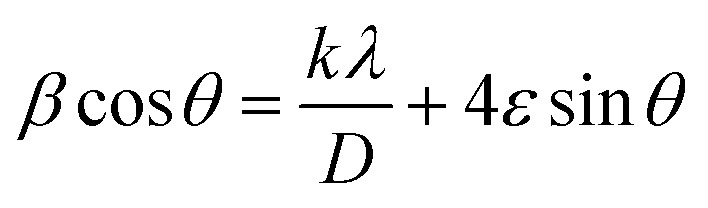
Here, for both equations, *D* = average crystallite size in nm, *λ* = wavelength of Cu X-ray = 0.15406 nm, *K* = crystallite shape factor = 0.9, *β* = full width at half-maxima in radian, *θ* = Bragg's angle in radian, and *ε* = microstrain.

**Table 1:** Average crystallite sizes and microstrains of undoped ZrO_2_ nanoparticles synthesized at different pH values

ZrO_2_ nanoparticles	2*θ* (degree) of the (101) peak	Average crystallite size (nm)		Microstrain, ×10^−3^
		Scherrer equation	W–H plot	
pH 1	30.26	8.00	14.13	4.36
pH 3	30.26	9.42	19.50	4.18
pH 7	30.14	18.25	30.21	3.19
pH 11	30.18	20.37	39.06	3.01

**Fig. 4:**
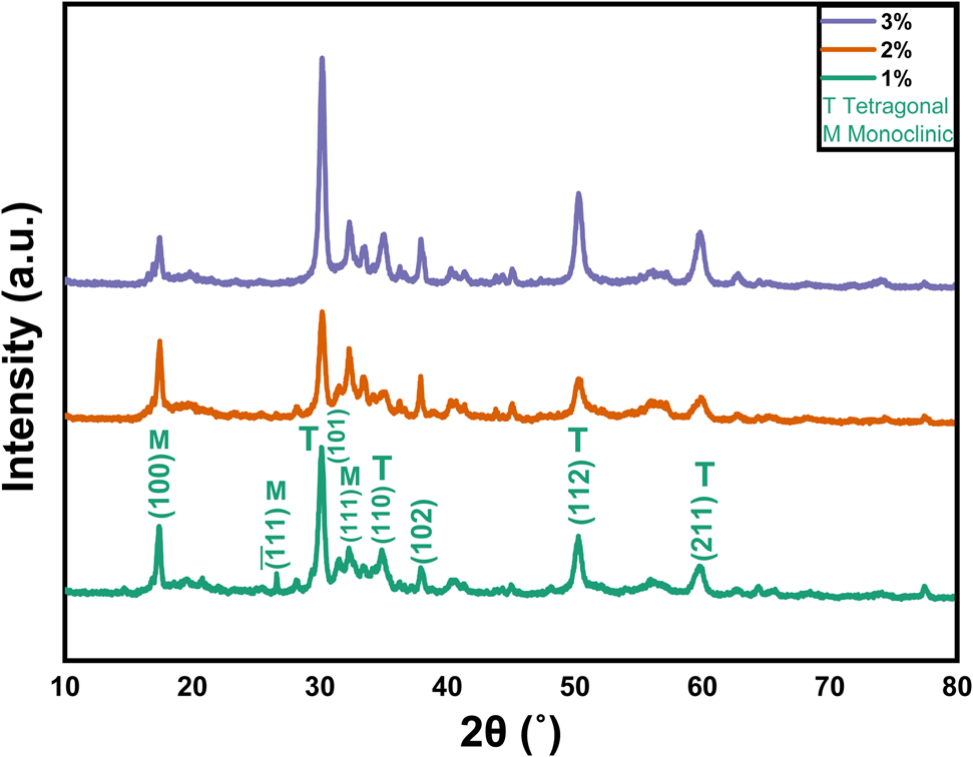
XRD patterns of 1%, 2%, and 3% Y–Ag doped ZrO_2_ nanoparticles.

**Fig. 5:**
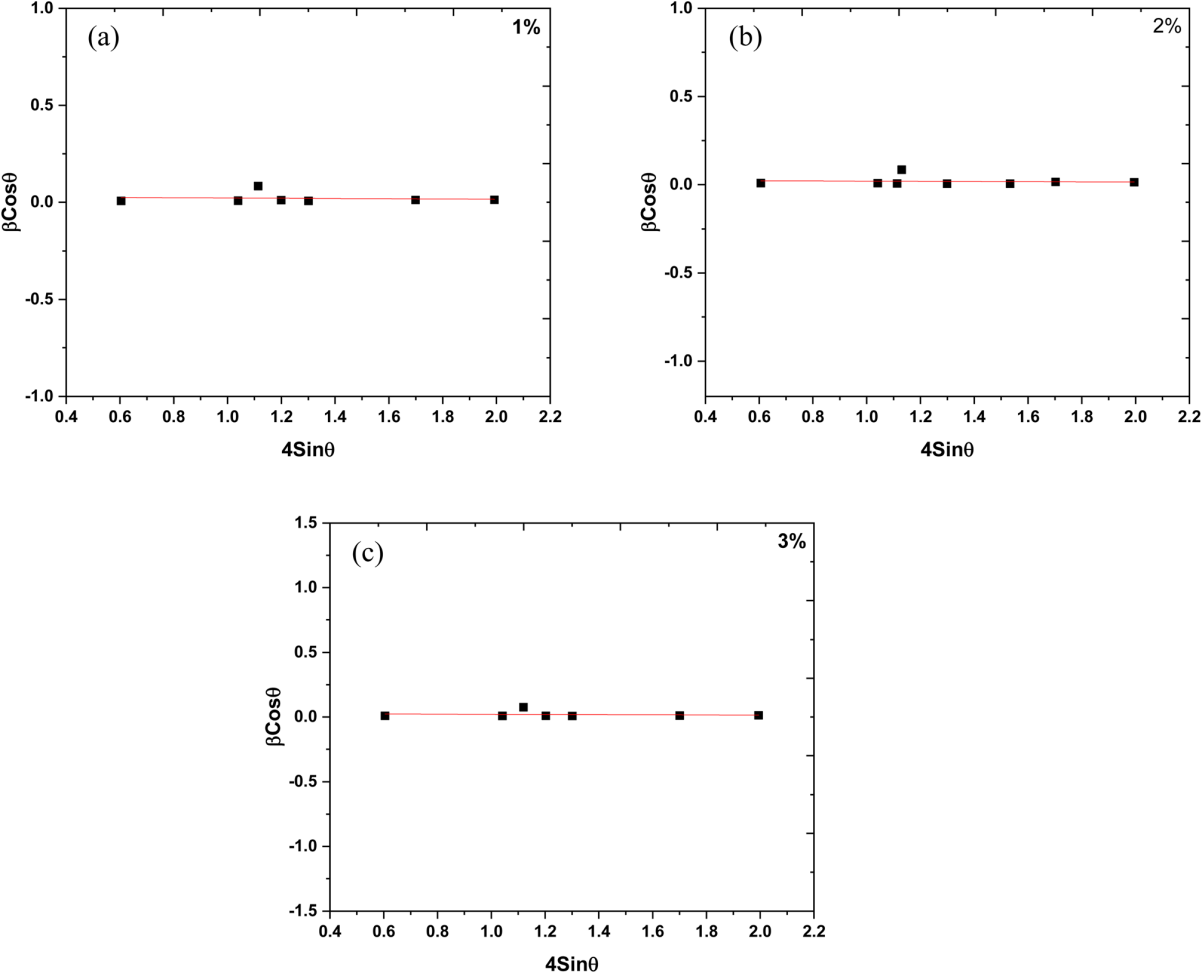
Williamson–Hall plots of (a) 1%, (b) 2%, and (c) 3% Y–Ag doped ZrO_2_ nanoparticles.

The XRD graphs of the 1%, 2%, and 3% Y and Ag codoped ZrO_2_ nanoparticles synthesized at pH 11 are presented in Fig. 4. The peaks at approximately 30.2°, 35.2°, 50.3°, and 60.1° correspond to the (101), (110), (112), and (211) planes, respectively, and confirm the presence of tetragonal zirconia (t-ZrO_2_, ICCD 01-079-1769). Additionally, the peaks at 17.4°, 28.2° and 31.5°, corresponding to the (100), (−111) and (111) planes, respectively, indicate a minor fraction of the monoclinic phase (m-ZrO_2_, ICDD 01-083-0941), confirming a multiphase structure. The substitution of Y^3+^, which has a smaller radius than Zr^4+^, compresses the lattice, generating oxygen vacancies that reduce the phase transformation temperature and stabilize t-ZrO_2_, while Ag^+^ induces lattice distortions favoring monoclinic ZrO_2_.^38–40^ The crystallite size and microstrain were determined by the Scherrer equation and Williamson–Hall plotting, respectively, as tabulated in Table 2. Fig. 5 shows the Williamson–Hall plots of 1%, 2%, and 3% Y and Ag codoped ZrO_2_ nanoparticles.

**Table 2:** Average crystallite sizes and microstrains of 1%, 2% and 3% Y–Ag doped ZrO_2_ nanoparticles

Nanoparticles	2*θ* (degree) of the (101) peak	Average crystallite size (nm)		Phase volume fraction (%)		Microstrain, ×10^−3^
		Scherrer equation	W–H plot	Tetragonal	Monoclinic	
ZrO_2_ codoped with 1% Y and Ag	30.2°	12.05	5.00	56.81	43.91	5.97
ZrO_2_ codoped with 2% Y and Ag	30.17°	14.81	5.5	45.94	54.06	5.31
ZrO_2_ codoped with 3% Y and Ag	30.17°	11.95	5.21	28.69	71.31	6.12

The volume fraction of the monoclinic phase (*v*_m_) was calculated by following the empirical formulas given in eqn (3) and (4), respectively:3
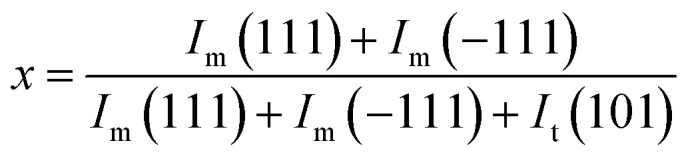
4
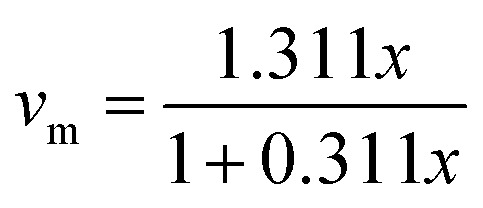
where *I*_m_ and *I*_t_ denote the intensities of monoclinic peaks and tetragonal peaks, respectively.

Due to the dopant's grain growth-inhibiting effect, the crystallite size is limited to 11–14 nm. At grain boundaries, Y^3+^ and Ag^+^ ions separate and pin them to stop excessive crystallite growth during synthesis.^39,40^ The observed negative microstrain is ascribed to lattice contraction resulting from the inclusion of smaller Y^3+^ ions, which produce compressive strain in the ZrO_2_ lattice.^41^ Ag^+^ doping somewhat offsets this effect, but the total lattice contraction caused by Y^3+^ doping predominates and generates negative microstrain.^40^

### 3.2. SEM analysis

**Fig. 6:**
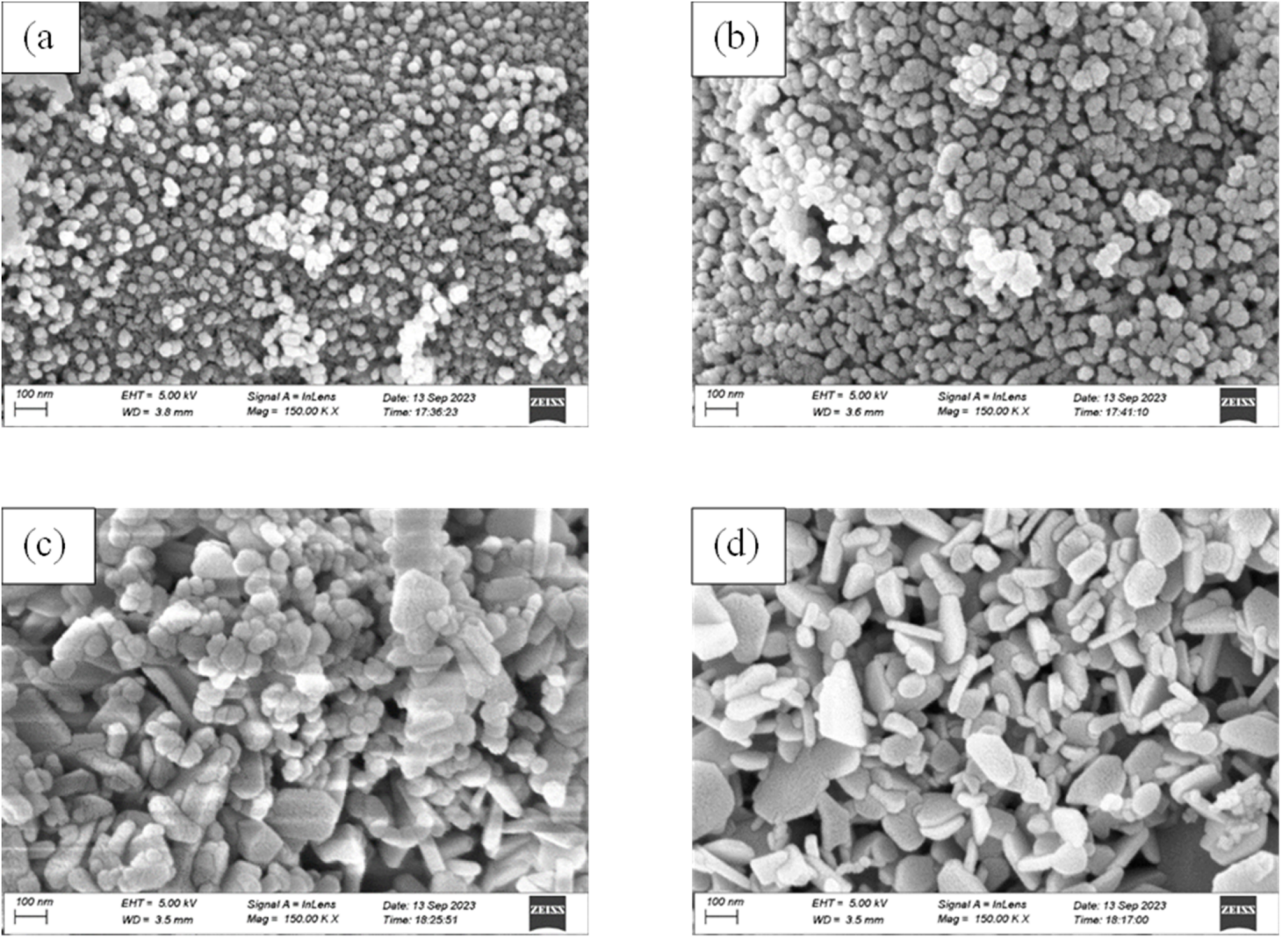
Scanning electron micrographs of (a) pH 1, (b) pH 3, (c) pH 7, and (d) pH 11 undoped ZrO_2_ nanoparticles.

**Fig. 7:**
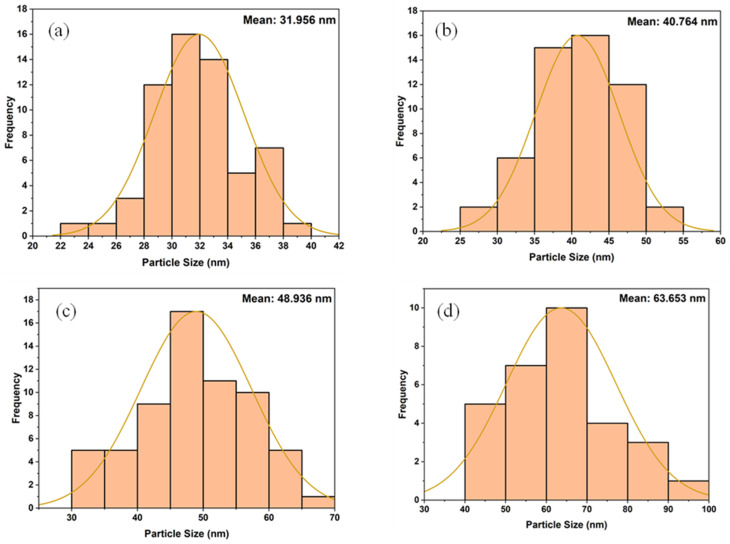
Particle size distribution curves of (a) pH 1, (b) pH 3, (c) pH 7, and (d) pH 11 undoped ZrO_2_ nanoparticles.

The morphology of the undoped ZrO_2_ synthesized at different pH levels of 1, 3, 7, and 11 was acquired using SEM analysis, and Fig. 6 illustrates the results. At pH 1 and 3, the shape of the particles was observed to be spherical with significant agglomeration, but with increasing pH, it appeared to have a rod-like to platelet-like morphology, with relatively better dispersion compared to lower pH.^42,43^ ImageJ software was used to measure the nanoparticles' sizes, and Fig. 7 shows the particle size distribution curves. At pH 1, pH 3, pH 7, and pH 11, the average particle size was 31.956 nm, 40.764 nm, 48.936 nm, and 63.653 nm, respectively.

Under acidic conditions (pH 1 and 3), spherical-shaped particles with significant agglomeration were observed. These were ascribed to fast hydrolysis and insufficient electrostatic stabilization at low pH, hence promoting uncontrolled particle growth and agglomeration.^44,45^ By contrast, samples synthesized at neutral and alkaline pH (7 and 11) showed a mostly rod-like to platelet-like morphology with better dispersion, presumably because of changed reaction kinetics and stronger surface charge repulsion at higher pH, hence lowering aggregation tendencies.^43^ From 31.956 nm (pH 1) to 63.653 nm (pH 11), the particle size increased methodically, implying that alkaline conditions favored Ostwald ripening and lower nucleation rates, thereby allowing increasing particle growth.^4,44,45^

**Fig. 8:**
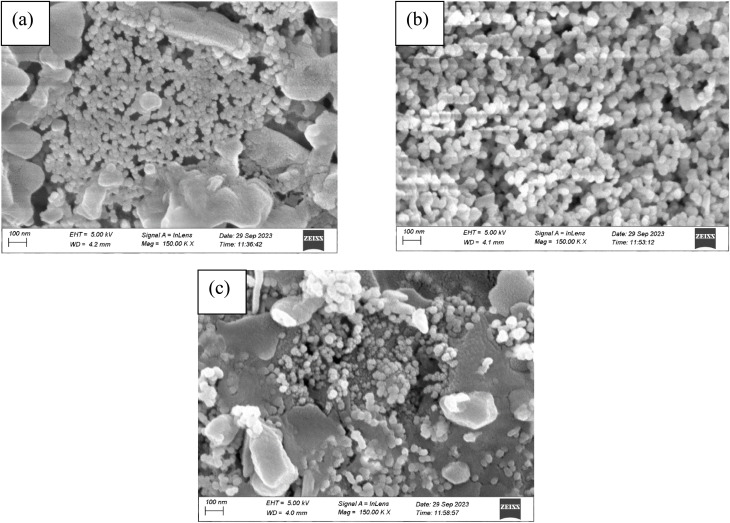
Scanning electron micrographs of (a) 1%, (b) 2%, and (c) 3% Y–Ag doped ZrO_2_ nanoparticles.

**Fig. 9:**
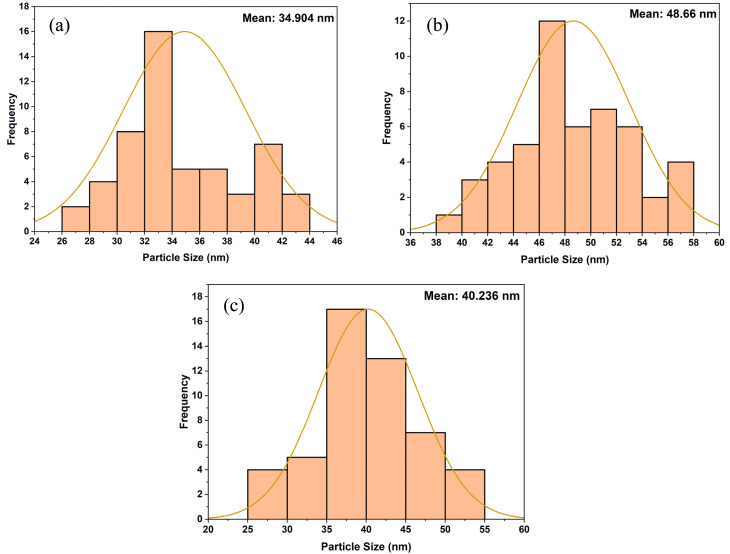
Particle size distribution curves of (a) 1%, (b) 2%, and (c) 3% Y–Ag doped ZrO_2_ nanoparticles.

The morphology of the 1%, 2%, and 3% Y and Ag codoped ZrO_2_ nanoparticles synthesized at pH 11 was analyzed using SEM, and the results are presented in Fig. 8. ImageJ software was used to determine the nanoparticles' size, and Fig. 9 shows the distribution curve of the nanoparticles. The nanoparticles mostly showed a spherical shape with significant agglomeration at 1% doping, thereby obtaining an average particle size of 34.904 nm. The particles became larger to 48.66 nm with a spherical shape and more clustering at 2% doping, presumably due to the stronger dopant interactions influencing growth dynamics. At 3% doping, the morphology changed to a mixture of spherical and irregular shapes with less aggregation compared to 2% doping, producing an average size of 40.236 nm. This range in the shape and dispersion implies that during synthesis, larger dopant concentrations affect the nucleation rates, surface energy, and interparticle interactions.^38,39^

The crystallite size of the nanoparticles calculated from XRD using the Scherrer equation ranged between 8 and 20 nm for undoped samples (pH 1–11) and 12 and 15 nm for Y, Ag codoped samples (Tables 1 and 2). However, the particle sizes measured from FESEM micrographs were comparatively larger, ranging from 31.95 nm at pH 1 to 63.65 nm at pH 11 for undoped ZrO_2_ and from 34.90 to 48.66 nm for codoped ZrO_2_ nanoparticles (Fig. 6–9). The larger particle sizes observed in SEM compared to XRD are attributed to the fact that XRD provides the average crystallite domain size, whereas SEM measures the physical particle size, which may include agglomerated crystallites and grain boundaries. Similar discrepancies between the crystallite size (XRD) and particle size (SEM) have been reported in earlier studies on metal oxide nanoparticles.^46^ Thus, the correlation indicates that each nanoparticle observed in SEM may consist of multiple crystallites, identified by XRD.

### 3.3. EDX analysis

**Fig. 10:**
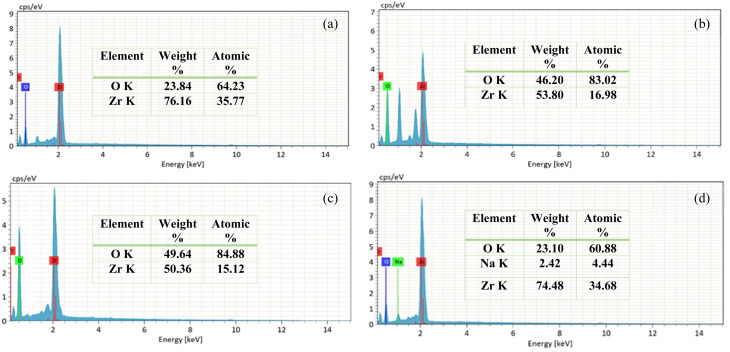
EDX spectra of (a) pH 1, (b) pH 3, (c) pH 7, and (d) pH 11 undoped ZrO_2_ nanoparticles.

The elemental composition of the synthesized undoped and codoped ZrO_2_ nanoparticles was determined by energy-dispersive X-ray spectroscopy (EDX). Fig. 10 shows the EDX spectra of the undoped ZrO_2_ synthesized at pH 1, 3, 7, and 11, where the characteristic Zr and O peaks are clearly visible, confirming the formation of pure ZrO_2_ nanoparticles. The presence of Na in the pH 11 sample is likely due to the basic synthesis environment, where Na^+^ ions from the alkaline medium may have been adsorbed during nanoparticle formation.

**Fig. 11:**
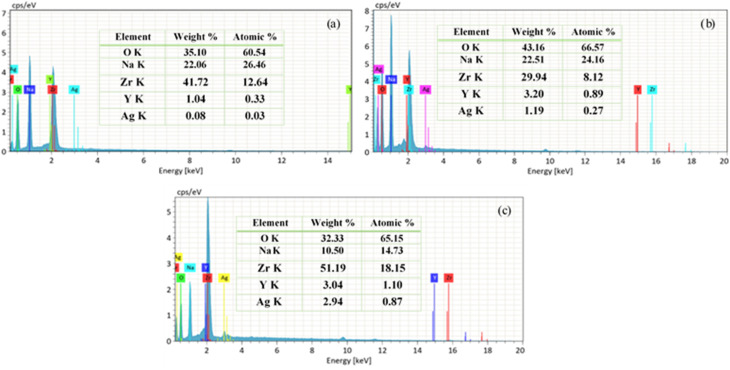
EDX spectra of (a) 1%, (b) 2%, and (c) 3% Y–Ag doped ZrO_2_ nanoparticles.

Fig. 11 shows the EDX spectra of Y, Ag codoped ZrO_2_ nanoparticles synthesized at pH 11, which confirms the presence of Y and Ag peaks alongside the primary Zr and O peaks.

The presence of distinct and well-defined peaks from Y and Ag indicates that both dopants have been successfully incorporated into the ZrO_2_ host lattice. Additionally, Na is observed in the doped samples, particularly at pH 11, which may be attributed to the basic synthesis environment. EDX analysis supports the XRD and SEM findings, validating the structural modifications induced by codoping. The successful incorporation of Y and Ag influences the material's composition, contributing to phase stability, morphology changes, and improved functional properties.

### 3.4. FT-IR analysis

**Fig. 12:**
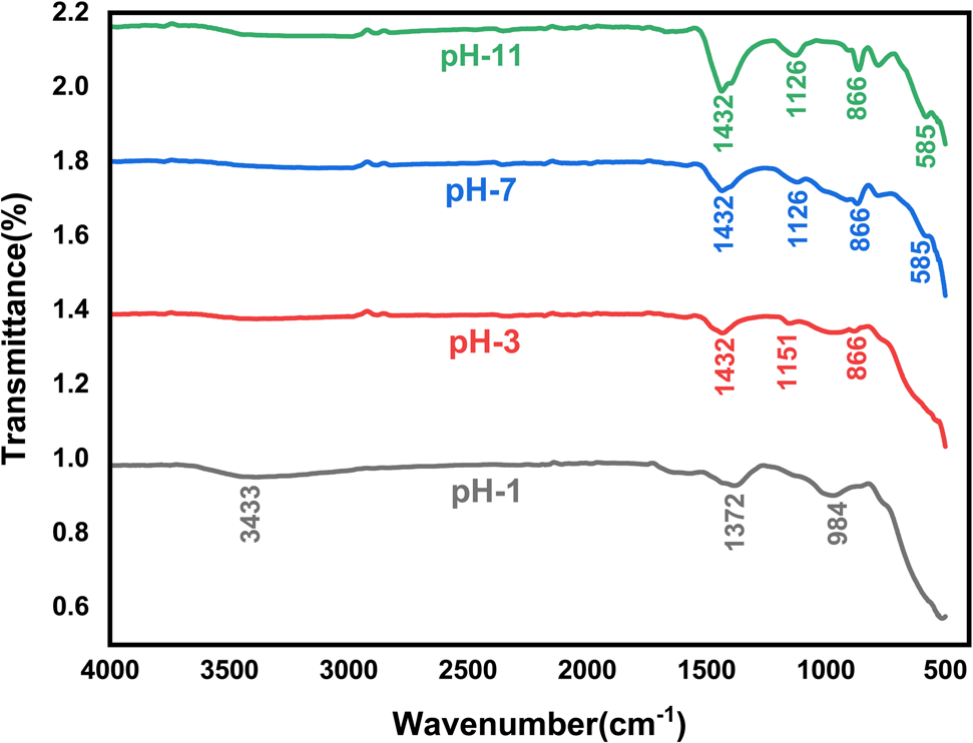
FT-IR spectra of pH 1, pH 3, pH 7, and pH 11 undoped ZrO_2_ nanoparticles.

The FT-IR spectra of the undoped ZrO_2_ nanoparticles synthesized at pH 1, pH 3, pH 7, and pH 11 are shown in Fig. 12. At pH 1, the peak observed at 3433 cm^−1^ corresponds to the O–H stretching vibration, which indicates the presence of hydroxyl groups due to incomplete condensation during the synthesis process. The presence of these hydroxyl groups can enhance hydrophilicity and influence antibacterial activity by interacting with the bacterial membrane.^47^ The characteristic peaks observed at 1432 cm^−1^, 1126 cm^−1^, 866 cm^−1^, and 585 cm^−1^ are attributed to carbonate groups (CO_3_^2−^), Zr–O–Zr asymmetric stretching vibrations and Zr–O stretching vibrations, respectively, which confirm the formation of ZrO_2_ nanoparticles.^48^

**Fig. 13:**
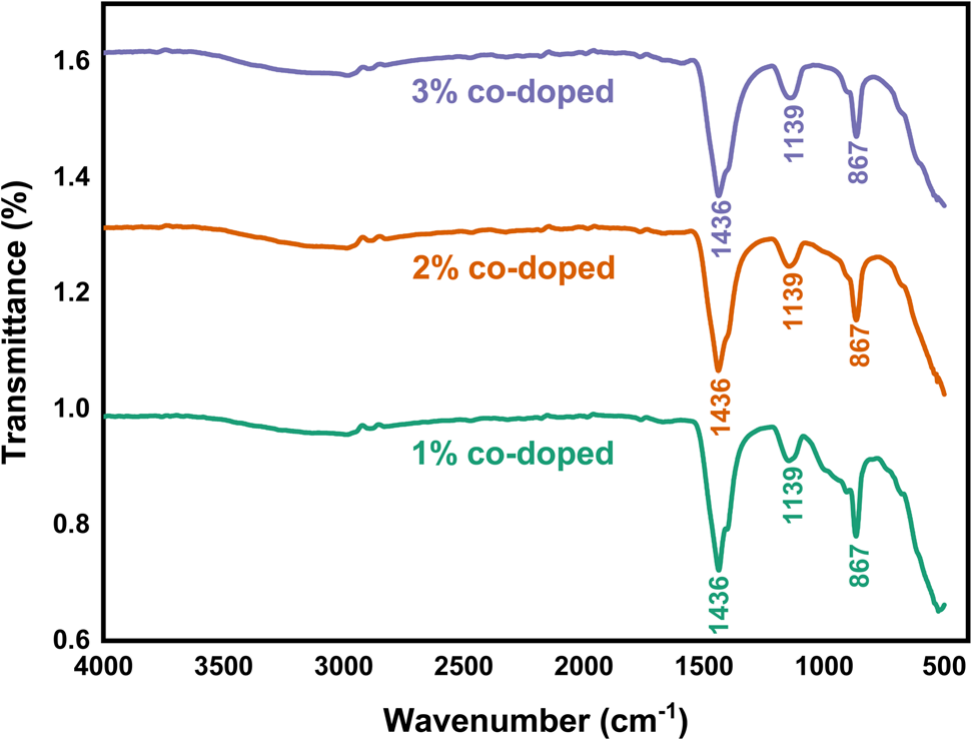
FT-IR spectra of 1%, 2%, and 3% Y–Ag doped ZrO_2_ nanoparticles.

The FT-IR spectra of 1%, 2%, and 3% Y and Ag codoped ZrO_2_ nanoparticles are shown in Fig. 13. The peaks at 1436 cm^−1^, 1139 cm^−1^, and 867 cm^−1^ are attributed to carbonate species (CO_3_^2−^), Zr–O–Zr stretching vibrations and Zr–O stretching vibration in the ZrO_2_ crystal structure, respectively.^49,50^ The preservation of these peaks across all doping levels suggests that the basic zirconia structure stays intact after Y and Ag inclusion. The small shift in the peak position suggests successful doping and possible modifications in lattice vibrations due to the interaction of Y^3+^ and Ag^+^ ions with the ZrO_2_ matrix.^38^ Y^3+^ and Ag^+^ dopants can change surface functional groups, hence improving antibacterial action *via* oxidative stress generation and membrane damage.^51,52^

### 3.5. Antibacterial activity

**Fig. 14:**
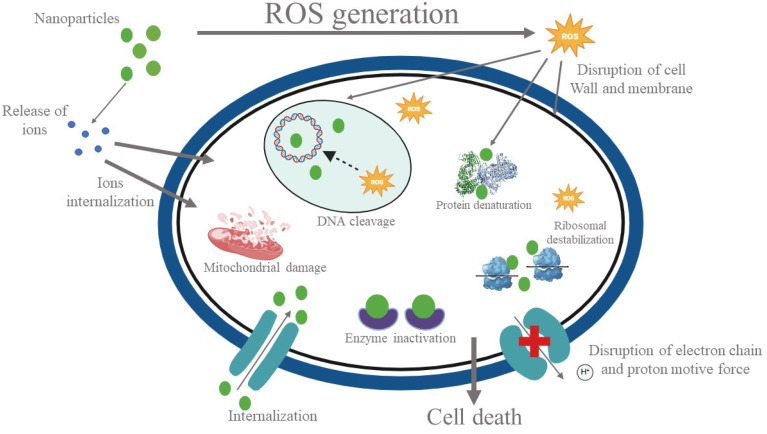
Antibacterial mechanism of ZrO_2_ nanoparticles.^54,55^

ZrO_2_ nanoparticles exhibit antibacterial activity through the generation of reactive oxygen species (ROS), membrane rupture, and cellular interference, resulting in bacterial cell death. Fig. 14 shows the antibacterial mechanism of ZrO_2_ nanoparticles. ZrO_2_ nanoparticles increase permeability and generate oxidative stress by interacting with bacterial membranes, releasing ions, all of which influence bacterial metabolism, ROS-induced protein breakdown, ribosome instability, and enzyme suppression. While also inducing DNA cleavage and replication suppression, ZrO_2_ nanoparticles influence mitochondrial activity and ATP production in *E. coli*. Against *E. coli* and *S. aureus*, ZrO_2_ nanoparticles show significant antibacterial properties.^53,54^

**Fig. 15:**
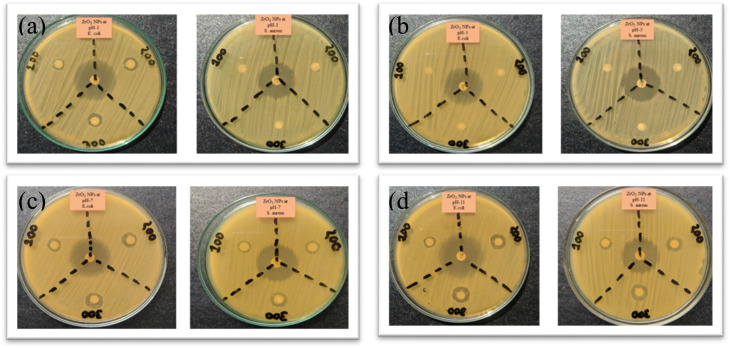
Antibacterial activity of undoped ZrO_2_ nanoparticles synthesized at (a) pH 1, (b) pH 3, (c) pH 7, and (d) pH 11 at 100, 200, and 300 μg mL^−1^ against *E. coli* and *S. aureus*.

The antibacterial activity of undoped ZrO_2_ nanoparticles was evaluated against *Escherichia coli* (Gram-negative) and *Staphylococcus aureus* (Gram-positive) using the disk diffusion method at varying concentrations (100, 200, and 300 μg mL^−1^) at different pH levels (1, 3, 7, and 11), and the results are presented in Fig. 15.

**Fig. 16:**
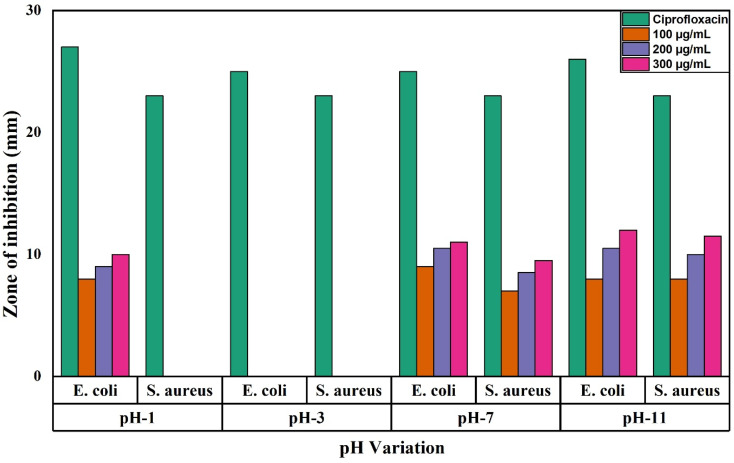
Diameter of the zone of inhibition of undoped ZrO_2_ nanoparticles synthesized at pH 1, pH 3, pH 7, and pH 11 against *E. coli* and *S. aureus*.

Ciprofloxacin was loaded as an antibiotic, and it is considered the drug of choice and used as a control for the treatment of bacterial infections. The zone of inhibition against pH variation is shown in Fig. 16.

At pH 3, nanoparticles reveal no inhibition zones at any concentration, likely because of aggregation and reduced surface charge stability, which weaken electrostatic interactions with bacterial membranes.^47,56^ By contrast, in a basic environment at pH 11, they show superior activity, with a 12-mm inhibition zone for *E. coli* at 300 μg mL^−1^, ascribed to an increased negative surface charge, better dispersion stability, and more reactive oxygen species (ROS) production.^20,57^ At pH 7, they show moderate efficacy, with an 11-mm inhibition zone for *E. coli*, where dose-dependent bacterial disruption is facilitated by an intermediate surface charge and partial nanoparticle adherence.^47^ While basic pH maximizes electrostatic and oxidative processes for improved bacterial membrane damage, the lack of activity at pH 3 emphasizes the vital importance of nanoparticle dispersibility and charge in antibacterial action.

**Fig. 17:**
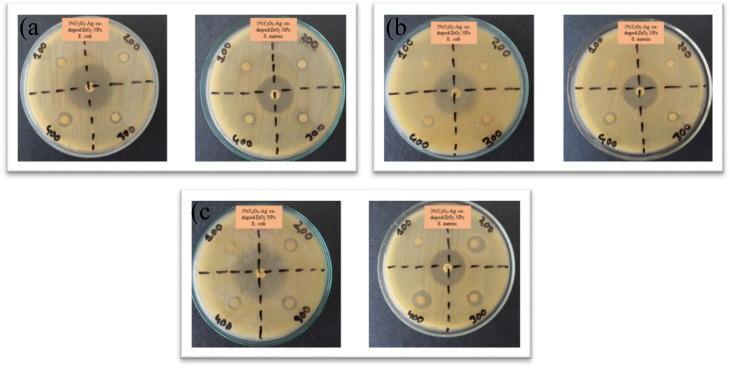
Antibacterial activity of (a) 1%, (b) 2%, and (c) 3% Y–Ag doped ZrO_2_ nanoparticles at 100, 200, 300, and 400 μg mL^−1^ against *E. coli* and *S. aureus*.

**Fig. 18:**
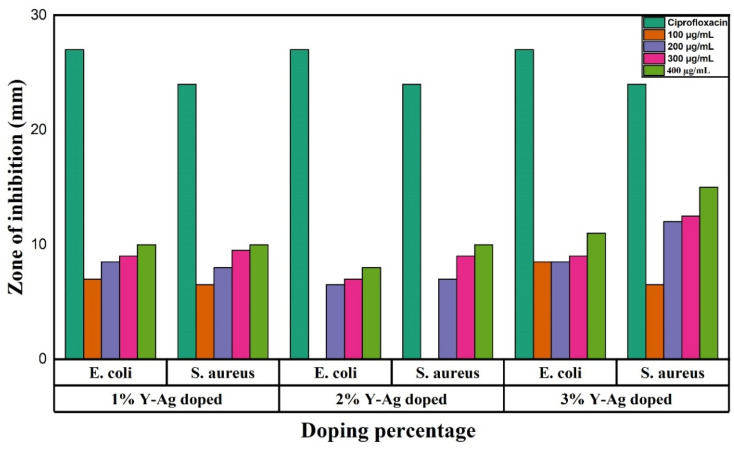
Diameter of the zone of inhibition of 1%, 2% and 3% Y–Ag doped ZrO_2_ nanoparticles against *E. coli* and *S. aureus*.

Fig. 17 presents the antibacterial activity of 1%, 2% and 3% Y and Ag codoped ZrO_2_ nanoparticles against *E. coli* and *S. aureus* at varying concentrations of 100, 200, 300, and 400 μg mL^−1^, respectively. At 1% codoping, the particles show a relatively lower antibacterial activity with the maximum inhibition zone of 10 mm for *E. coli* and 10 mm for *S. aureus* at a 400 μg mL^−1^ concentration. By contrast, at 2% codoping, they show moderate activity, and at 100 μg mL^−1^, no inhibition is observed, suggesting a concentration threshold for effective bacterial suppression. The zone of inhibition against the doping percentage is shown in Fig. 18. However, the excellent antibacterial activity is observed at 3% codoping, with the highest inhibition zone of 11 mm for *E. coli* and 15 mm for *S. aureus* at 400 μg mL^−1^. The higher doping percentage-improved antibacterial activity can be ascribed to the synergistic action of Ag^+^ ions and Y, which promotes oxidative stress, membrane damage, and protein inactivation in bacterial cells.^58^ The dose-dependent rise in the inhibition zones further supports the concentration-dependent bactericidal activity of the nanoparticles.

The antibacterial trends align with recent findings that nano-ZrO_2_ suppresses the growth of common pathogens, with performance strongly influenced by the particle size and surface state.^59^ Compared with Ag/ZrO hybrids, our Y, Ag codoped samples show an expected improvement relative to undoped ZrO_2_ but remain below antibiotic controls, which is consistent with reports that Ag incorporation yields the largest bactericidal gains.^60^ The influence of yttria observed here also agrees with the literature, showing that Y stabilization can modulate antibacterial response through phase and compositional changes.^61^

### 3.6. Toxicity analysis

**Fig. 19:**
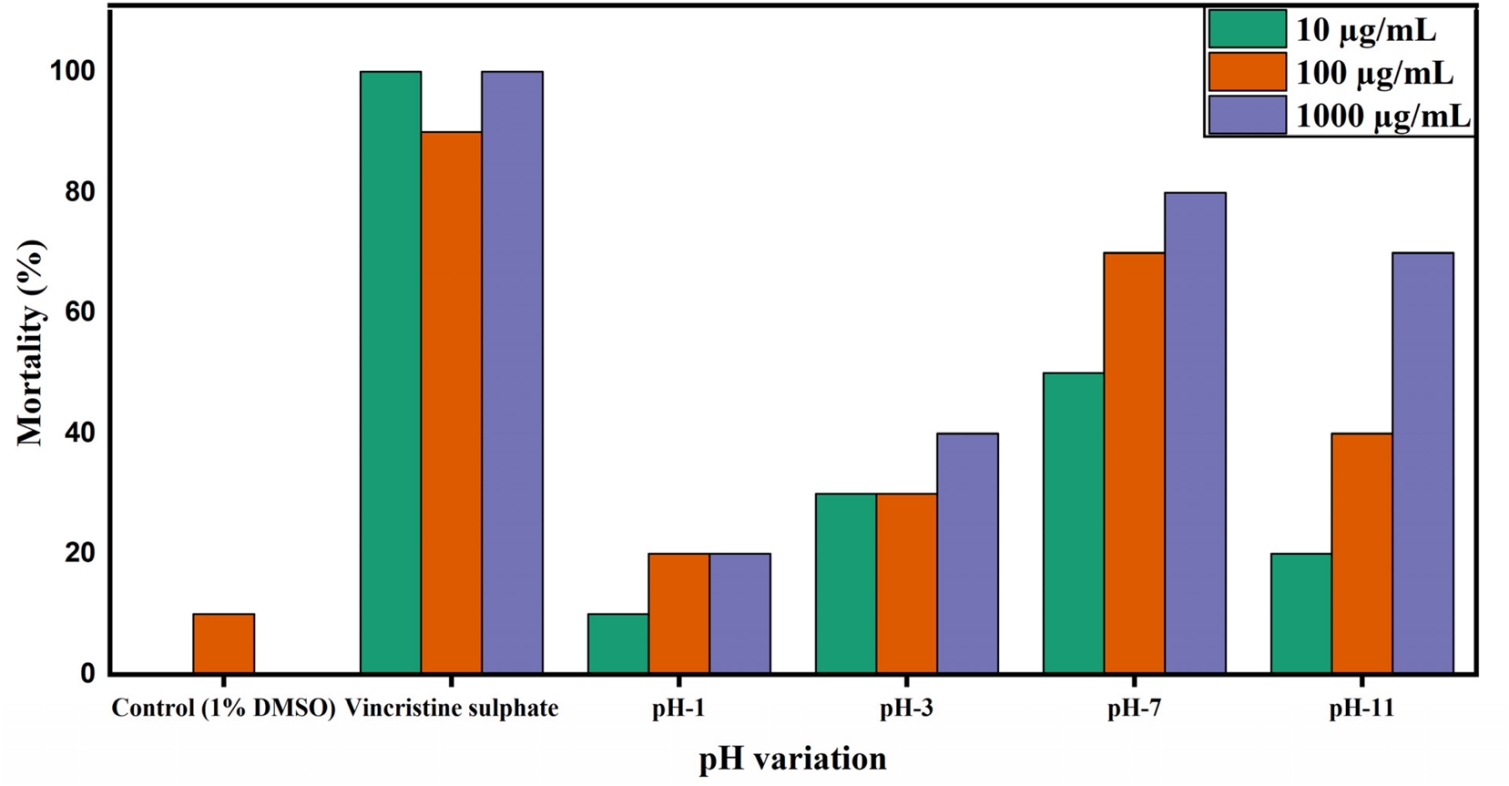
Mortality percentage of undoped ZrO_2_ nanoparticles at pH 1, pH 3, pH 7, and pH 11 in the brine shrimp lethality assay.

The toxicity of ZrO_2_ nanoparticles was inspected using the brine shrimp lethality assay, and the corresponding responses are shown as percentage mortality after treating with different concentrations of ZrO_2_ nanoparticles ranging from 10 to 1000 μg mL^−1^ for 24 h. Fig. 19 shows the percentage of mortality against pH variation.

The contrast control (1% DMSO) consistently exhibited minimal mortality across all concentrations, indicating negligible toxicity from the solvent. The positive control, vincristine sulphate, induced near-complete mortality at all concentrations, confirming its strong cytotoxic potential. At pH 1 and pH 3, the ZrO_2_ nanoparticles displayed relatively low mortality (<40%), even at the highest concentration, suggesting limited toxicity under highly acidic conditions. At neutral pH (pH 7), mortality increased substantially, reaching up to 80% at 1000 μg mL^−1^. This enhanced cytotoxic response may be due to better particle dispersion and surface charge characteristics at physiological pH, which facilitate stronger interactions with cellular components and the disruption of biological membranes.^62,63^ At pH 11, moderate toxicity was observed, with mortality levels rising alongside the concentration, reaching 70% at 1000 μg mL^−1^.

**Fig. 20:**
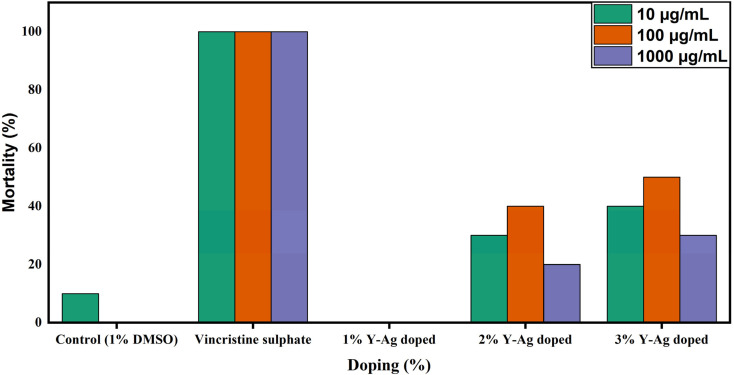
Mortality of 1%, 2%, and 3% Y–Ag doped ZrO_2_ nanoparticles in the brine shrimp lethality assay.

Fig. 20 presents the percentage of mortality at different doping percentages in the brine shrimp lethality assay. The 1% doped sample exhibited no mortality at any tested concentration, indicating excellent biocompatibility. This lack of toxicity may be attributed to the surface stabilization imparted by the Y–Ag codoping, which likely reduces aggregation and improves dispersion in biological media.^33,62^ Higher doping levels (2% and 3%) resulted in increased mortality, particularly at elevated concentrations. The 2% doped sample showed mortality rates up to 40%, while the 3% doped sample reached a 50% mortality rate at 100 μg mL^−1^. Interestingly, a slight reduction in mortality was observed at 1000 μg mL^−1^ for the 2% doped sample, potentially due to nanoparticle settling or altered interaction dynamics at higher concentrations.

## 4. Conclusion

In this study, undoped and codoped ZrO_2_ nanoparticles were successfully synthesized *via* a sucrose-assisted sol–gel approach. By altering their pH and doping concentration, the effects of the doping elements and pH were observed. The effect of undoped and codoped ZrO_2_ nanoparticles was tested for antibacterial activity and toxicity. According to XRD data, the nanoparticles formed a tetragonal structure at lower pH, formed a monoclinic structure at higher pH, and formed a multiphase structure after codoping. The size of the nanoparticles and the existence of chemical components within them were revealed by SEM and EDX, which did not contradict the revised results from XRD analysis. The results revealed that increasing the pH from 1 to 11 led to an increase in the crystallite size from 8 nm to 20 nm and the particle size from 31.96 nm to 63.65 nm. For 1%, 2%, and 3% doped nanoparticles, the sizes were 34.904 nm, 48.66 nm and 40.236 nm. FTIR data confirmed the presence of relevant bond and metal–oxygen vibrations in the synthesized particles. The results of the antibacterial activity analysis against *E. coli* and *S. aureus* revealed that the best activity was observed at pH 11 for undoped particles and 3% Y–Ag codoped particles. The brine shrimp lethality assay was used to examine the toxicity of undoped and codoped ZrO_2_ nanoparticles, and it was shown that the material was less cytotoxic at lower pH values (1 and 3) and that the toxicity increased as the doping concentration increased. Therefore, the results of this study show that the synthesized ZrO_2_ nanoparticles are a suitable candidate for biomedical applications.

## Author contributions

Mehedi Hasan Jasim: writing – original draft, methodology, investigation, conceptualization. Md. Iqbal Hossain: writing – original draft, investigation, data curation, formal analysis. Yasfir Mahmud: writing – original draft, methodology, investigation, conceptualization. A. K. M. Ahsanul Habib: writing – review & editing, supervision, methodology, investigation, data curation, formal analysis, conceptualization. Moumita Tasnim Meem: writing – review & editing, supervision, resources, project administration, methodology, conceptualization.

## Conflicts of interest

The authors declare that there are no conflicts of interest regarding the publication of this article.

## Data Availability

Data will be made available on request.
